# Enhancing COVID-19 Detection: An Xception-Based Model with Advanced Transfer Learning from X-ray Thorax Images

**DOI:** 10.3390/jimaging10030063

**Published:** 2024-02-29

**Authors:** Reagan E. Mandiya, Hervé M. Kongo, Selain K. Kasereka, Kyamakya Kyandoghere, Petro Mushidi Tshakwanda, Nathanaël M. Kasoro

**Affiliations:** 1 Mathematics, Statistics and Computer Science Department, University of Kinshasa, Kinshasa XI P.O. Box 190, Democratic Republic of the Congo; reagain.mandiya@unikin.ac.cd (R.E.M.); herve.kongo@unikin.ac.cd (H.M.K.); nathanael.kasoro@unikin.ac.cd (N.M.K.); 2 Artificial Intelligence, Big Data and Modeling Simulation Research Center (ABIL), Kinshasa XI P.O. Box 190, Democratic Republic of the Congo; 3 Institute of Smart Systems Technologies, University of Klagenfurt, 9020 Klagenfurt am Wörthersee, Austria; 4 Department of Electrical and Computer Engineering, University of New Mexico, Albuquerque, NM 87131, USA; pmushidi@unm.edu

**Keywords:** COVID-19, Xception, X-ray images, neural network, medical imaging, transfer learning

## Abstract

Rapid and precise identification of Coronavirus Disease 2019 (COVID-19) is pivotal for effective patient care, comprehending the pandemic’s trajectory, and enhancing long-term patient survival rates. Despite numerous recent endeavors in medical imaging, many convolutional neural network-based models grapple with the expressiveness problem and overfitting, and the training process of these models is always resource-intensive. This paper presents an innovative approach employing Xception, augmented with cutting-edge transfer learning techniques to forecast COVID-19 from X-ray thorax images. Our experimental findings demonstrate that the proposed model surpasses the predictive accuracy of established models in the domain, including Xception, VGG-16, and ResNet. This research marks a significant stride toward enhancing COVID-19 detection through a sophisticated and high-performing imaging model.

## 1. Introduction

The relentless global impact of the COVID-19 pandemic has underscored the critical importance of early and accurate detection in safeguarding public health, mitigating economic repercussions, and ensuring the long-term well-being of communities worldwide [1,2,3]. Early detection allows diseases to be diagnosed at a stage when they are more likely to respond to treatment, reduces morbidity and mortality, prevents complications and consequences, and lowers healthcare costs. Timely detection not only informs effective patient care but also serves as a linchpin in elevating long-term survival rates, emphasizing the pressing need for innovative diagnostic methodologies. It, therefore, seems important to think about setting up systems capable of facilitating the early detection of disease to contribute to public healthcare management.

Over the years, medical imaging has emerged as an indispensable tool for the early detection, monitoring, and post-treatment follow-up of diseases. From the inception of computer-aided diagnostic systems in the early 1980s to the contemporary era of advanced artificial intelligence (AI) applications, the trajectory of medical image analysis has evolved significantly. While early approaches focused on sequential processing and mathematical modeling, the advent of AI, inspired by the human brain’s learning mechanisms, has ushered in a new era of sophisticated diagnostic systems [4,5].

Machine learning, a cornerstone of this transformative paradigm, empowers software applications to enhance predictive accuracy without explicit programming. At the forefront of global health concerns, the ongoing COVID-19 pandemic, caused by the severe acute respiratory syndrome coronavirus 2 (SARS-CoV-2), demands innovative solutions for effective patient screening. Despite the widespread adoption of the reverse transcription polymerase chain reaction test, its limited positivity rate and inability to differentiate SARS-CoV-2 from other respiratory infections underscore the urgent need for alternative screening methods [6,7,8].

Artificial intelligence models for rapid disease detection are systems that employ machine learning, image analysis, or natural language processing techniques to identify individuals infected with SARS-CoV-2, the virus responsible for COVID-19. These models can rely on various types of data, such as radiological images, antigen tests, reported symptoms, or genomic data, offering speed, accuracy, user-friendliness, and cost reduction. They play a crucial role in screening suspected cases, guiding patients to appropriate care, monitoring disease progression, and controlling virus spread.

However, detecting and accurately distinguishing between different strains of SARS-CoV-2 poses a formidable challenge in the landscape of disease detection. The evolving nature of the virus, coupled with its propensity for genetic mutations, introduces complexities that demand innovative solutions. One crucial hurdle lies in the scarcity of sufficient, reliable, and representative data for training and validating models, especially for emerging virus variants. These variants may necessitate adaptations or updates to existing models, highlighting the need for continuous vigilance and adjustment. Additionally, the variability in model performance across diverse contexts, populations, environments, and usage protocols further accentuates the intricacy of the task. The ethical, legal, and social dimensions of deploying these models also contribute to the multifaceted challenges, encompassing issues of privacy, data protection, responsibility, transparency, explainability, security, reliability, and trust. The gravity of these challenges necessitates a comprehensive and principled approach to model development and usage, emphasizing the urgency of addressing these intricacies for the advancement of disease detection strategies.

To tackle these challenges, adherence to principles and best practices for the development and use of artificial intelligence models for rapid disease detection is crucial. Guidelines proposed by organizations like the World Health Organization (WHO) [9], the Organisation for Economic Co-operation and Development (OECD), and the European Commission provide valuable insights.

Furthermore, fostering collaboration and data, knowledge, and experience sharing among stakeholders involved in the fight against COVID-19, including researchers, healthcare professionals, policymakers, industry professionals, and citizens, is essential. This collaborative approach can significantly contribute to overcoming the challenges posed by the rapidly evolving viral threat and enhancing the effectiveness of AI models in disease detection.

The key contributions of this paper include the proposition and validation of an innovative model, synthesized from the strengths of existing architectures and enriched through transfer learning. Experimental results demonstrate superior predictive accuracy compared to benchmark models. By advancing the state of the art in COVID-19 detection, this research significantly contributes to global efforts to revolutionize patient care pathways and bolster long-term survival rates. In the next section, an introduction to transfer learning is provided to facilitate a smooth transition into the discussion on its relevance to the proposed deep learning model.

The remainder of this paper is organized as follows. Section 2, “Materials and Methods”, presents the methodology used for our innovative deep learning model for automated COVID-19 detection, and the dataset and experimental setup are also outlined. Section 3, “Results”, presents the detailed findings from the validation experiments. In Section 4, “Discussion”, the implications, challenges, and limitations of the results are comprehensively discussed, along with a comparison with related works. Section 5, “Conclusions”, concludes the paper with a synthesis of the key insights and future directions for the proposed model.

## 2. Materials and Methods

### 2.1. Xception Model

Xception, short for “Extreme Inception”, is a state-of-the-art deep learning architecture proposed by François Chollet in 2017 [10]. It represents a significant advancement in convolutional neural network (CNN) design, particularly tailored for image classification tasks [11]. At its core, Xception embodies a fundamental departure from conventional CNN architectures, introducing a novel approach to convolution operations.

Traditional CNNs rely on standard convolutional layers to extract features from input images. These layers apply a set of learnable filters across the entire input volume, producing feature maps that capture spatial patterns. However, this approach often leads to an excessive number of parameters, resulting in computational inefficiency and increased risk of overfitting.

In contrast, Xception introduces depth-wise separable convolutions, a concept borrowed from the Inception family of architectures [12]. Depth-wise separable convolutions decompose the standard convolution operation into two distinct stages: depth-wise convolution and point-wise convolution.

Let *F* represent the input feature map, *K* denote the kernel (or filter), and *S* signify the stride length. The depth-wise convolution operation is defined as:

(1) $$F_{ij}^{\prime }=\sum_{m,n}F_{\left(i\cdot S+m)(j\cdot S+n\right)}\times K_{mn}$$

where $F_{ij}^{\prime }$ represents the output feature map and $K_{mn}$ denotes the corresponding element of the kernel. By performing convolutions independently across each channel of the input feature map, depth-wise convolutions significantly reduce computational complexity while preserving spatial information.

Following the depth-wise convolution, point-wise convolutions are applied to integrate information across channels. This operation is expressed as:

(2) $$F_{ij}^{\prime }=\sum_{k}F_{ij}\times K_{k}$$

where $K_{k}$ represents the *k*-th element of the point-wise kernel. By incorporating both depth-wise and point-wise convolutions, Xception achieves a remarkable balance between computational efficiency and expressive power.

In addition to its architectural innovations, Xception employs other techniques, such as batch normalization and ReLU activations, to enhance model stability and convergence speed [13]. These elements collectively contribute to Xception’s exceptional performance in image classification tasks, making it a preferred choice for various applications, including medical image analysis.

In the subsequent sections, we delve deeper into the integration of Xception within our proposed deep learning paradigm, elucidating its role in revolutionizing automated disease detection from medical imaging data.

### 2.2. Transfer Learning

Transfer learning is a powerful concept in machine learning that leverages knowledge gained from one task to improve performance on a different but related task [14]. In the context of deep learning and neural networks, transfer learning involves using pre-trained models on large datasets and fine-tuning them for specific tasks. This approach is particularly beneficial when the target task has limited labeled data.

Transfer learning can be conceptualized as follows: let *S* denote the source domain, *T* denote the target domain, P(S) denote the probability distribution of the source domain, P(T) denote the probability distribution of the target domain, *X* denote the input space, and *Y* denote the output space. The model’s objective is to learn a mapping f:X→Y that performs well on *T* based on the knowledge acquired from *S*.

Transfer learning encompasses various strategies, such as feature extraction and fine-tuning. Feature extraction involves using the pre-trained model’s early layers as generic feature extractors and appending task-specific layers for the target task. Fine-tuning, on the other hand, refines the entire model on the target task by adjusting the weights of all layers while retaining the knowledge gained from the source task.

### 2.3. Transfer Learning Framework: Feature Extraction, Encoding, Decoding, and Feature Generation

In the transfer learning phase, we introduce various layers, including a feature extraction module that enables us to address the expressiveness issue resulting from different locations and times of image capture. This module precedes encoding and decision making for effective preprocessing. The methodology encompasses a feature-encoding module, a decoding module, and a feature-generation segment. This approach is both sequential and parallel, enhancing the overall efficiency of the process.

In our proposed model for COVID-19 detection, transfer learning plays a pivotal role in overcoming the challenges associated with the evolving nature of the SARS-CoV-2 virus. By leveraging pre-existing knowledge from a large dataset, the model can effectively learn relevant features for distinguishing between different strains, enhancing its accuracy and robustness in the face of emerging variants. The subsequent sections delve into the specifics of our novel model and the experimental validation conducted to demonstrate its superior predictive accuracy compared to established models.

In the quest for a groundbreaking solution to the crucial problem at hand, we introduce a novel deep learning paradigm meticulously designed to redefine the landscape of automated COVID-19 detection from X-ray thorax images. Our proposed model seamlessly integrates the robust Xception architecture for pattern recognition, offering a transformative approach to enhance diagnostic accuracy in the realm of healthcare.

### 2.4. Model Architecture

**Figure 1:**
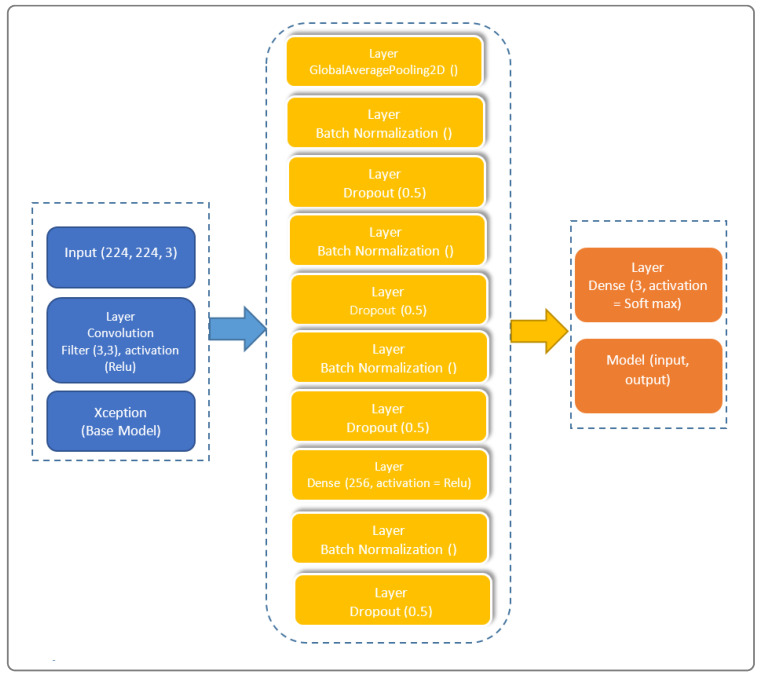
Architecture of the proposed Xception-Enhanced Transfer Learning Model. In blue is the feature extraction block, in yellow is the transfer learning block, and in orange is the classification block.

The architectural prowess of our proposed model is illustrated in Figure 1, encapsulating the fusion of cutting-edge techniques for feature extraction, transfer learning, and classification. In the spirit of innovation, we have strategically organized the model into distinctive blocks, each contributing to the overall efficacy. Our model’s architecture, meticulously crafted to train on existing data and predict outcomes for new individuals, stands as a testament to the sophistication required in the healthcare domain.

Our model is subdivided into three blocks. The first contains the basic Xception model. The second contains a “GloabalAveragePooling2D” layer followed by four “BatchNormalization” layers, which provide data belonging to the same scale. This makes the neural network easier to train. Normalization, therefore, consists of formatting the input data to facilitate the machine learning process. These layers are separated by a dropout layer, with a rate of 0.5 to minimize the risk of overlearning, and a dense layer with 256 units linking all the layers of the network. The third and final block consists of a dense layer with a “softmax” activation function.

### 2.5. Layer Model

Considering the combination of two functions ϖ and φ to produce a new function ϖ×φ, we define the convolution model in Equation (3)

(3) $$\left(\varpi \times \varphi \right)\left(x\right)=\int_{-\infty }^{\infty }\varpi \left(t\right)\varphi \left(x-t\right)dt$$

**Figure 2:**
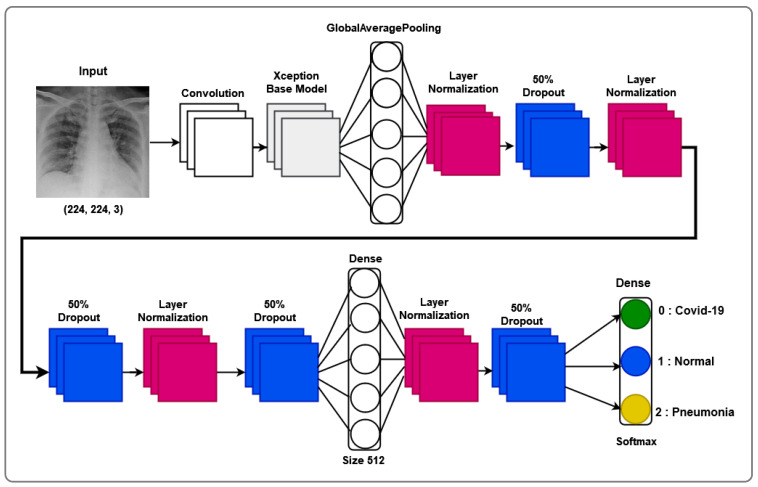
Layer model of our proposed Xception-Enhanced Transfer Learning Model.

To this, we add its decomposition into two distinct stages: a depth convolution, applying a spatial filter of size J×J to each input channel, and a point convolution, then applying a 1×1 filter to all output channels, allowing for the reduction or increase in the number of channels, ensuring depth-separable convolution. An Xception block is necessary in our approach, as it constitutes a basic unit of the Xception approach. To this end, we consider a succession of depth-separable convolution layers, followed by a batch normalization layer and an activation function, as illustrated by the layered model shown in Figure 2.

To add transfer learning, let us consider any task denoted by T as an image classification function defined by Equation (4), as follows:

(4) T=(V,W)

where V is the input space and W is the output space.

To predict the images, we then define *f*, a function that associates a numerical value with each element of V or W. To have a function that approximates the function to be learned, using adjustable parameters, we denote *M* as an image classification model such that M(v) defines the model prediction for image v∈V.

Considering that the source and target tasks share certain common features, which can be captured by the model, we then define two subsets as follows:

The features are given by $f_{1},f_{2},...,f_{n}\in F$ and the parameters by $p_{1},p_{2},...,p_{m}\in P$.

We have $\exists F\subset f:V_{S}\cup V_{C}\to R,\exists P\subset R,\forall f\in F,\forall p\in P$, $M_{S}\left(v_{S}\right)=f\left(v_{S},p\right)\mathrm{and}\;M_{C}\left(v_{C}\right)=f\left(v_{C},p\right)$, where $M_{S}$ is the source model associated with the source task $T_{S}=\left(V_{S},W_{S}\right)$ and $M_{C}$ is the target model used by the target task $T_{C}=\left(V_{C},W_{C}\right)$.

Figure 2 illustrates our proposed layer model, showcasing the intricate composition that underlies the model’s ability to navigate the complexities of X-ray thorax images. The layer model emphasizes the interplay between feature extraction, transfer learning, and classification layers, providing a comprehensive insight into the neural architecture’s depth and sophistication.

### 2.6. Integration of Xception Model: Rationale and Advantages

The decision to incorporate the Xception network into our model stems from its multifaceted advantages. Xception, evolving from Inception modules within convolutional neural networks, strategically positions itself as an intermediate step between conventional convolution and the depth-separable convolution operation. This distinctive characteristic empowers our model with unparalleled adaptability and expressive capacity, crucial for intricate nuances in COVID-19 pattern recognition within X-ray thorax images.

Our proposed Xception-Enhanced Transfer Learning Model represents a pioneering stride toward revolutionizing the diagnostic landscape in healthcare. By harnessing the strengths of Xception and seamlessly integrating them into our deep learning paradigm, we anticipate a paradigm shift in the accuracy and efficiency of automated COVID-19 detection. The subsequent sections delve into the experimental validation, results, and discussions, providing a comprehensive narrative of the model’s performance and its potential impact on the broader healthcare domain.

Furthermore, while numerous image analysis methods, such as the YOLO model, demonstrate high performance in computer vision, our specific study opts for the Xception model due to its exceptional performance in the medical domain. Unlike other models, Xception introduces depth-wise separable convolutions, enhancing its capabilities.

Xception transforms the original Inception-V3 block by expanding it and replacing various convolution operations (1 × 1, 5 × 5, 3 × 3) with a single 3 × 3 convolution followed by a 1 × 1 convolution. This modification aims to effectively regulate computational complexity. Additionally, unlike Inception, which applies ReLU non-linearities after convolution operations, depth-wise separable convolutions are generally implemented without non-linearities.

The choice of Xception for our methodology is grounded in its prowess in medical applications and its innovative architectural modifications, contributing to improved computational efficiency without compromising performance.

### 2.7. Tools and Technologies Used

The realization of our work leveraged cutting-edge tools and technologies. On the hardware front, an HP Probook computer with a Windows 10 operating system, 64-bit architecture, Intel(R) Core i7-9700F CPU @ 3.00GHz, and 16 GB of RAM played a pivotal role. The software toolkit used in the experiment included TensorFlow 1.5.3, Keras 2.15.0, Scikit-learn 1.2.2, Scikit-image 0.19.3, Python 3.11.6, and Flask 3.0.2.

### 2.8. Dataset

**Figure 3:**
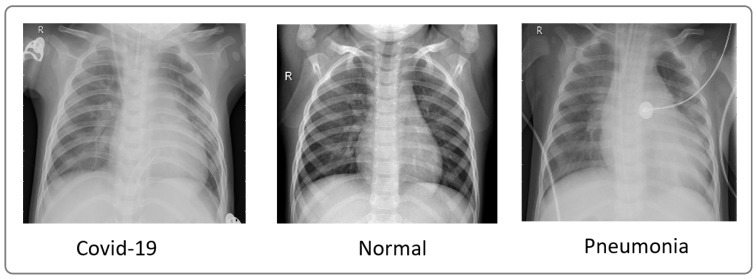
Sample of images from the dataset used [16].

To evaluate our model, we utilized X-ray images from COVID-19 patients sourced from multiple datasets, including hospital data related to the COVID-19 outbreak [15,16] and Kaggle data [16]. We considered two datasets. The first one comprised 4050 images, with 3000 images for training and 1050 images for testing. The second one comprised 6378 images, with 4878 images for training and 1500 images for testing. These datasets included images from confirmed COVID-19 patients, normal individuals, and pneumonia patients. Figure 3 showcases a sample of X-ray images from these datasets.

Table 1 displays the distribution by class of the first dataset with 3000 images (training), including 2400 images for training and 600 images for validation.

Table 2 presents the class distribution of the second dataset with 4878 images (train), including 3902 training images and 976 images for validation.

## 3. Results

### 3.1. Xception-Enhanced Transfer Learning Model

**Figure 4:**
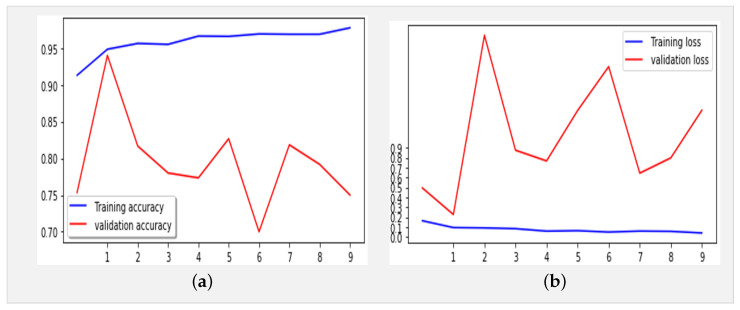
(**a**) Accuracy and (**b**) loss for the Xception model using the X-ray images dataset (10 epochs).

Figure 4 displays the performance results obtained by the Xception model using the same dataset (Table 1) over 10 epochs during training.

The proposed model, with 21,412,395 parameters, was first trained for 10 epochs utilizing the Adam optimizer (learning rate: 0.0001) and categorical cross-entropy for error computation. The key optimization techniques included LearningRateSchedule and ReduceLROnPlateau to dynamically adjust the learning rates. The model achieved an impressive accuracy of 96% in training and 97% in validation, with error rates of 0.07% and 0.06%, respectively. The classification report of our proposed model, as shown in Table 3, highlights exceptional performance across all classes (COVID-19, normal, and pneumonia), with precision and recall scores consistently exceeding 92.9%. The overall F1-score of 97.6% underscores the model’s robustness in accurately classifying X-ray images, marking a significant advancement in the field of COVID-19 detection.

**Figure 5:**
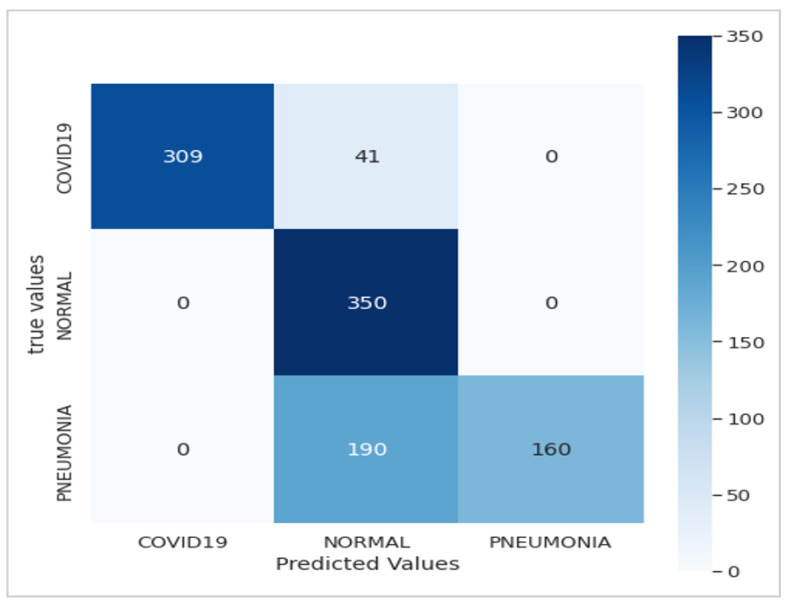
Confusion matrix of the Xception model.

**Figure 6:**
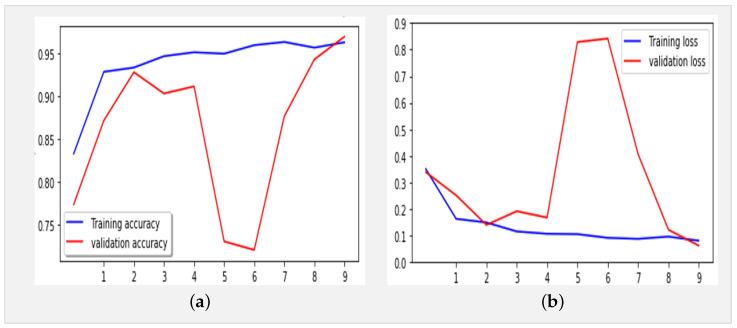
(**a**) Accuracy and (**b**) loss for the Xception-Enhanced Transfer Learning Model using the X-ray images dataset (10 epochs).

Figure 5 presents the confusion matrix of the Xception model, showcasing its robust performance. Figure 6 summarizes the relationship between accuracy and loss for our proposed model during training and validation. These results were obtained using the dataset summarized in Table 1 over 10 epochs during training.

**Figure 7:**
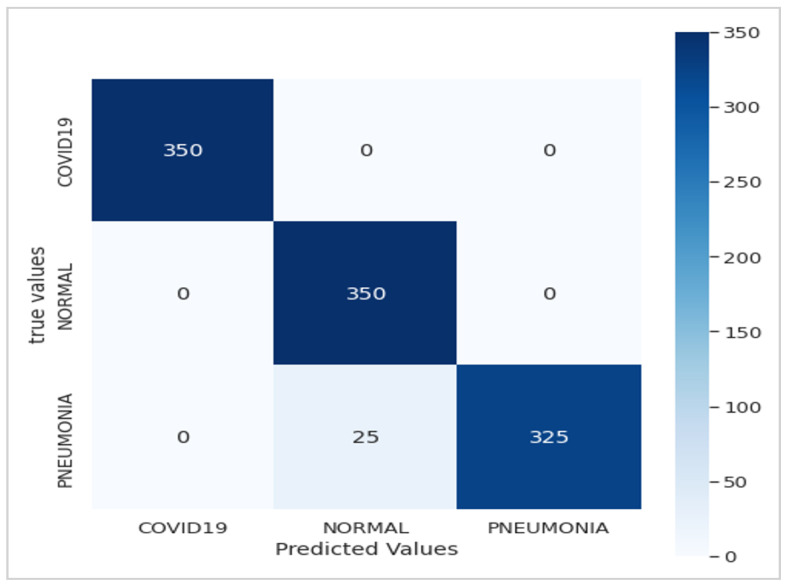
Confusion matrix of the Xception-Enhanced Transfer Learning Model (10 epochs).

We trained our model using a dataset comprising 3902 images distributed across three classes (COVID, normal, and pneumonia) for training data and 1500 for testing, necessitating a methodical approach (Table 2). Firstly, it was crucial to preprocess the images by resizing them to (224, 224, 3), normalizing the pixel values, and splitting them into distinct sets for training and validation. Subsequently, we compiled the model by defining the appropriate loss function (such as cross-entropy) and optimizer (Adam) to guide network learning. While we acknowledge that the typical input size for Xception models is 299 × 299 × 3, we resized our X-ray images to 224 × 224 × 3 during preprocessing for compatibility with our dataset. This resizing was conducted while considering the balance between computational efficiency and preserving essential diagnostic information. Despite the dimension reduction, standard resizing techniques were employed to maintain the integrity of the images. The model was trained on the training data over multiple iterations (epochs), totaling 21 epochs, where it adjusted its weights to minimize loss and enhance performance. Following training, it was crucial to evaluate the model on test data to assess its ability to generalize to new, unseen data. By analyzing metrics such as accuracy, precision, and recall for each class, we can assess the model’s performance and identify areas for improvement. Finally, adjustments can be made to the model, such as tuning hyperparameters or augmenting data, to enhance its performance (Figure 7).

**Figure 8:**
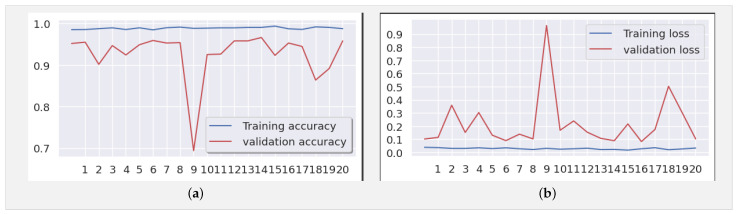
(**a**) Accuracy and (**b**) loss for the Xception-Enhanced Transfer Learning Model using the X-ray images dataset (21 epochs).

**Figure 9:**
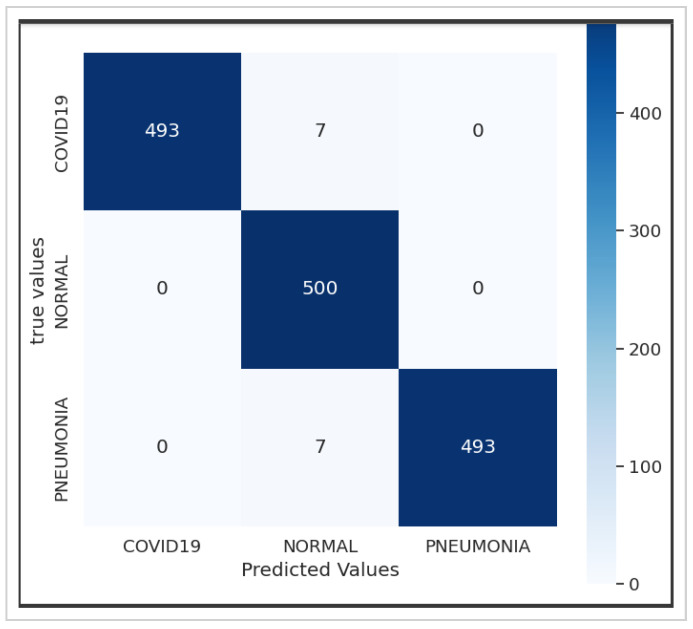
Confusion matrix of the Xception-Enhanced Transfer Learning Model (21 epochs).

Figure 8a,b depict the training and validation accuracy, respectively, after training our model with 80% of the data used, along with the error rates for 80% of the training data. The corresponding confusion matrix, as illustrated in Figure 9, visualizes the model’s performance, displaying the number of correct and incorrect predictions made by the model for each class compared to the true labels of the data. This matrix is useful for evaluating accuracy, recall, specificity, and other model performance metrics, helping in the identification of classification errors and areas where the model can be improved. As shown in Table 4, by increasing the size of the dataset and the number of epochs, our model demonstrates even higher performance.

**Figure 10:**
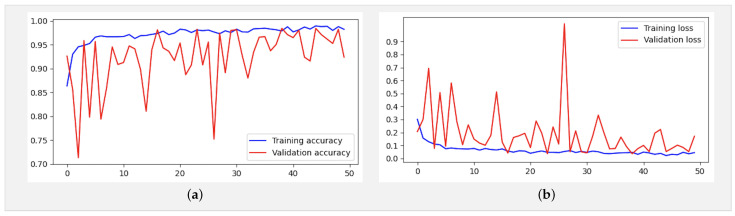
(**a**) Accuracy and (**b**) loss for the Xception-Enhanced Transfer Learning Model using the X-ray images dataset (50 epochs).

In addition, an analysis of Figure 8a,b reveals deviations starting from the ninth epoch. To address this, we extended the training duration from 21 to 50 epochs. As depicted in Figure 10a,b, while occasional deviations persisted at certain epochs, the overall trend stabilized as training progressed toward the 50th epoch. These observations underscore the robustness and reliability of our findings, offering a deeper insight into the behavior and performance characteristics of the model.

### 3.2. Benchmark Models

To benchmark our model, we selected ResNet50, VGG-16, and Xception, presenting a comparative summary in Table 5.

## 4. Discussion

### 4.1. Evolution of Neural Network Architectures

In contemporary computer vision applications, convolutional neural networks (CNNs) stand as a cornerstone, offering a versatile architecture characterized by alternating convolution and subsampling layers. The structural arrangement of these layers, complemented by innovations such as batch normalization [13] and dropout [17], significantly impacts the network’s performance. Tailoring the layout of CNN layers plays a pivotal role in architectural design, influencing the overall efficacy of the model [18,19].

A myriad of pattern recognition models has emerged in the recent literature, each leveraging distinct neural network architectures. Pioneering models like LeNet [20], AlexNet [21], GoogleNet [22], ResNet [23,24], DenseNet [5], and Xception have demonstrated the evolution of neural network design. In the quest for enhanced performance, researchers have explored innovative strategies, combining data and prior knowledge in hybrid models [25]. Noteworthy examples include VGG, which analyzes the impact of depth on accuracy through a network with up to 19 layers [26]. Despite its success, VGG’s computational complexity, with 138 million parameters, hinders deployment on resource-constrained systems.

In addressing this limitation, GoogleNet, also known as Inception-V1, introduced a block concept, leveraging convolutional transformations at multiple scales while minimizing computational cost [27]. This approach replaces conventional layers with small blocks, reducing the parameter count from 138 million to 4 million. The model’s heterogeneity, however, necessitates customization for each network. ResNet, proposed in [13], pioneered residual learning, featuring an architecture of up to 152 layers organized into residual blocks. ResNet exhibits reduced computational complexity and lower error rates in classification tasks.

The evolution of neural network architectures continued with Xception, introduced in [10] to enhance Inception-V3 modules through depth-separable convolutions. Departing from the original Inception-V3 block, Xception expands and replaces diverse convolution operations with a single 3×3 convolution followed by a 1×1 convolution, effectively regulating computational complexity. Notably, Xception has outperformed benchmarks such as VGG-16, ResNet, and Inception-V3 in traditional classification challenges, offering a resource-efficient alternative for clinical model development due to its commendable trade-off between resource consumption and accuracy [10]. We provide an insightful overview of key models, namely ResNet50, VGG-16, and Xception, encompassing their architectural intricacies, intended purposes, performance metrics, and inherent limitations. The summarized information is presented in Table 6, offering a comprehensive comparison to contextualize our proposed model’s advancements.

### 4.2. Performance Evaluation and Benchmarking

In the evaluation and benchmarking results presented in Table 5, our innovative model, the Xception-Enhanced Transfer Learning Model, demonstrated superior accuracy compared to its counterparts. To further strengthen the credibility of our findings, we calculated the confidence intervals for key performance metrics including accuracy, precision, recall, and F1-score. These intervals offer valuable insights into the variability of our model’s performance, enhancing the robustness of our results. With a confidence level of 95%, the calculated 95% confidence interval for the accuracy of our model was approximately 0.991 ± 0.00477, indicating a range from 0.98623 to 0.99577. The inclusion of these confidence intervals alongside our results provides a comprehensive assessment of the stability and reliability of our model’s performance. Notably surpassing the highly acclaimed Xception model, our innovation has set a new standard in the field. Figure 4, Figure 6 and Figure 8 vividly depict the superior accuracy of our model compared to Xception.

Utilizing the dataset showcased in Figure 3, our model achieved an exceptional precision of over 98.8%, surpassing that of ResNet50 (60%), Xception (86.74%), and VGG-16 (92%), as highlighted in Table 5. The confusion matrices presented in Figure 5, Figure 7 and Figure 9 further underscore the superior performance of our model compared to Xception.

This stellar performance can be attributed to the strategic incorporation of transfer learning, specifically the synergy between Xception and our proposed model. The integration of transfer learning enhances the accuracy and overall efficacy of our model. The resource-efficient nature of our model, coupled with its ability to achieve robust performance with a modest amount of labeled training data, distinguishes it as the pinnacle of current machine learning models.

## 5. Conclusions

In this study, we introduced the Xception-Enhanced Transfer Learning Model as a novel approach for precise COVID-19 detection from X-ray images. Leveraging the power of transfer learning, our model has demonstrated exceptional performance across various metrics, surpassing established benchmarks and setting a new standard in diagnostic accuracy.

Our model’s success is evidenced by its outstanding performance compared to other models, as summarized in Table 5. With a training accuracy of 96% and a validation accuracy of 97%, our model consistently outperforms ResNet50, VGG-16, and even the baseline Xception model. Furthermore, our model exhibits impressive recall and precision rates of 97% and 98.8%, respectively, highlighting its robustness in correctly identifying COVID-19 cases while minimizing false positives.

By harnessing transfer learning, our approach not only achieves superior accuracy but also addresses key challenges in model development. The utilization of pre-trained models, such as Xception, significantly reduces the need for extensive labeled data, making our model both resource-efficient and scalable for deployment in real-world settings. Moreover, the integration of transfer learning enhances our model’s adaptability to diverse datasets and medical imaging tasks, paving the way for future advancements in diagnostic methodologies.

Our findings underscore the transformative potential of advanced machine learning techniques in combating global health crises. The Xception-Enhanced Transfer Learning Model represents a paradigm shift in COVID-19 detection, offering enhanced diagnostic capabilities that can significantly impact patient care pathways. Beyond its immediate implications for COVID-19 diagnosis, our model lays the foundation for the development of innovative diagnostic tools in healthcare, promising improved outcomes and better management of infectious diseases.

## Figures and Tables

**Table 1:** Data distribution used for training (training images: 80%; validation images: 20%).

	COVID-19	Pneumonia	Normal	Total
Training	823	763	814	2400
Validation	177	237	186	600
Total				3000

**Table 2:** Second data distribution used for training (training images: 80%; validation images: 20%).

	COVID-19	Pneumonia	Normal	Total
Training	1308	1303	1291	3902
Validation	335	323	318	976
Total				4878

**Table 3:** Classification report of our proposed model over 10 epochs based on the data presented in Table 1. COVID-19 is class 0, normal is class 1, and pneumonia is class 2.

Class	Precision	Recall	F1-Score	Support
0	1.000	1.000	1.000	350
1	0.933	1.000	0.966	350
2	1.000	0.929	0.963	350
Accuracy			0.976	1050
Macro Avg	0.978	0.976	0.976	1050
Weighted Avg	0.978	0.976	0.976	1050

**Table 4:** Classification report of our proposed model over 21 epochs based on the data presented in Table 2. COVID-19 is class 0, normal is class 1, and pneumonia is class 2.

Class	Precision	Recall	F1-Score	Support
0	1.000	0.986	0.993	500
1	0.973	1.000	0.986	500
2	1.000	0.986	0.993	500
Accuracy			0.991	1500
Macro Avg	0.991	0.991	0.991	1500
Weighted Avg	0.991	0.991	0.991	1500

**Table 5:** Comparative table of the results obtained for different models.

Model	ResNet50	VGG-16	Xception	Our Model
Epoch	10	10	10	10
Training accuracy	51.66%	64.54%	73.51%	96%
Validation accuracy	47.83%	71.83%	73.68%	97%
Training loss	0.9142	0.4405	0.1217	0.0705
Validation loss	0.8384	0.4594	0.3272	0.0621
Test accuracy	66.66%	90.33%	78%	97.7%
Test loss	0.7486	0.128	0.4672	0.0575
Recall	59%	91%	78%	97%
Precision	60%	92%	86.74%	98.8%

**Table 6:** Overview of selected models (ResNet50, VGG-16, Xception, and our proposed model), including the models’ architectures, purposes, performance, and limitations.

Model	Architecture	Purpose	Performance	Limitations	Refs.
ResNet50	A deep CNN model that uses a residual architecture. It uses residual blocks to enable deep learning without a significant reduction in performance.	Often used for image classification, object detection, and semantic segmentation tasks. Also used in image recognition, fraud detection, medical research, and video surveillance.	Renowned for its exceptional performance in solving image classification problems. It has won numerous computer vision challenges thanks to its ability to learn highly discriminating representations.	Can be more complex to train and require more computing power because of its depth.	[23]
VGG-16	A CNN model that consists of 16 convolution and pooling layers. It uses convolutions of size 3×3 with strides of 1 and pooling of size 2×2 to extract image features.	Used for image classification, object detection, and automatic generation of image descriptions and artistic research.	A powerful model for image classification. Relatively heavier in terms of the number of parameters.	Has a simpler architecture compared to ResNet50 and Xception but can suffer from overfitting when used with smaller datasets.	[26]
Xception	A CNN model that uses an architecture based on depth-wise convolutions to reduce the number of parameters and improve computational efficiency.	Used for image classification, object detection, and semantic segmentation tasks, as well as emotion recognition, medical image classification, and anomaly detection.	Improves the performance of models based on depth convolutions but may require more computational resources.	Efficient in terms of the number of parameters, but it can be slower to train due to the complexity of its architecture.	[10]
Our Model	Subdivided into three blocks. The first contains the basic Xception model. The second contains a global average pooling 2D layer, followed by four batch normalization layers. The third consists of a dense layer with a softmax activation function.	Our model can be used for image classification, object detection and semantic segmentation tasks, pattern recognition, and medical image classification.	Xception improves the performance of models based on depth convolutions. Therefore, we added a transfer learning block to train the model and further improve Xception’s basic performance.	Our model is subject to some of Xception’s limitations. However, thanks to its third block, it improves the high-training-time problem suffered by Xception.	-

## Data Availability

The data used in this study are openly available at https://www.kaggle.com/datasets/jtiptj/chest-xray-pneumoniacovid19tuberculosis (accessed on 29 October 2023). The Python source code developed for the Xception-Enhanced Transfer Learning Model can be found at https://github.com/sedjokas/Enhanced-Transfer-Model (accessed on 1 February 2024).

## Abbreviations

The following abbreviations are used in this manuscript:

AI	Artificial Intelligence
CNN	Convolutional Neural Network
CPU	Central Processing Unit
RAM	Random Access Memory
VGG	Visual Geometry Group
